# Supplementary material for: Yiai Fuzheng decoction inhibits triple-negative breast cancer by remodeling the immune microenvironment
Source: Front Immunol. 2025 Sep 30;16:1615631. doi: 10.3389/fimmu.2025.1615631 (PMC12518410; doi:10.3389/fimmu.2025.1615631)
Supplement: Supplementary file 2 [file DataSheet1.zip › Supplementary File 2/Treatment/C_vs_D_vs_H/06_Pathway_Analysis/Pathway_Analysis/Sig_Pathways_Name/Glycine, serine and threonine metabolism.pdf]

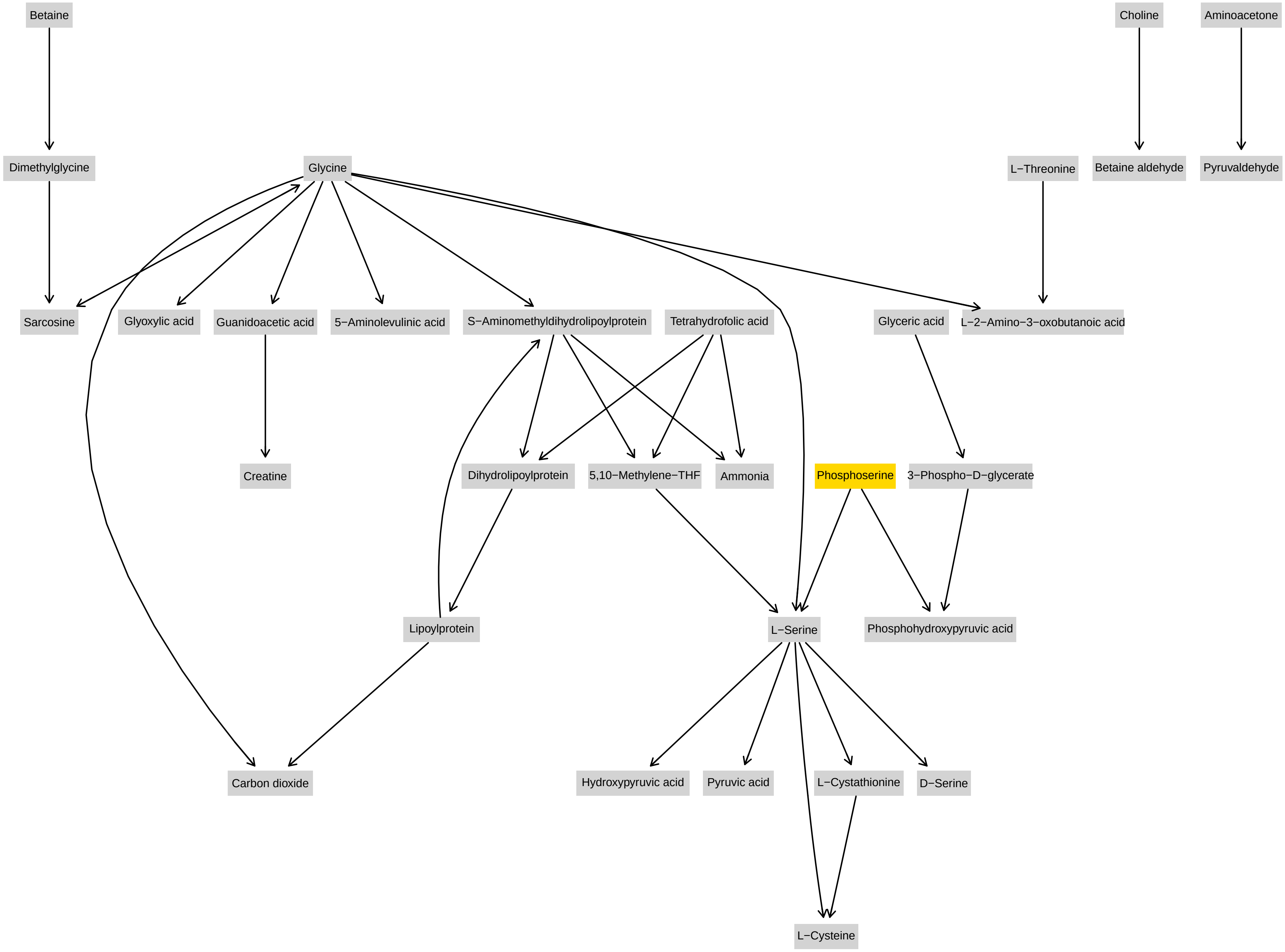
Betaine
Choline
Aminoacetone
Dimethylglycine
Glycine
L−Threonine
Betaine aldehyde
Pyruvaldehyde
Sarcosine
Glyoxylic acid
Guanidoacetic acid
5−Aminolevulinic acid
S−Aminomethyldihydrolipoylprotein
Tetrahydrofolic acid
Glyceric acid
L−2−Amino−3−oxobutanoic acid
Creatine
Dihydrolipoylprotein
5,10−Methylene−THF
Ammonia
Phosphoserine
3−Phospho−D−glycerate
Lipoylprotein
L−Serine
Phosphohydroxypyruvic acid
Carbon dioxide
Hydroxypyruvic acid
Pyruvic acid
L−Cystathionine
D−Serine
L−Cysteine
